# Integrated Sampling Approaches Enhance Assessment of Saproxylic Beetle Biodiversity in a Mediterranean Forest Ecosystem (Sila National Park, Italy)

**DOI:** 10.3390/insects16080812

**Published:** 2025-08-06

**Authors:** Federica Mendicino, Francesco Carlomagno, Domenico Bonelli, Erica Di Biase, Federica Fumo, Teresa Bonacci

**Affiliations:** Department of Biology, Ecology and Earth Science, University of Calabria, 87036 Rende, CS, Italy; federica.mendicino@unical.it (F.M.); domenico.bonelli@unical.it (D.B.); ericadibia@gmail.com (E.D.B.); fumofede@gmail.com (F.F.); teresa.bonacci@unical.it (T.B.)

**Keywords:** saproxylic beetles, biodiversity assessment, deadwood, forest monitoring, integrated sampling, Mediterranean forests, forest ecosystem health

## Abstract

Saproxylic beetles are key bioindicators of forest ecosystem quality and play essential roles in deadwood decomposition and nutrient cycling. However, their populations are increasingly threatened by habitat fragmentation, deadwood removal, and climate-driven environmental changes. For this reason, an integrated sampling method can increase the detection of species with varying ecological traits. We evaluated the effectiveness of integrative sampling methodologies to assess saproxylic beetle diversity within Sila National Park, a Mediterranean forest ecosystem of high conservation value, specifically in two beech forests and four pine forests. The sampling methods tested included Pan Traps (PaTs), Malaise Traps (MTs), Pitfall Traps (PTs), Bait Bottle Traps (BBTs), and Visual Census (VC). All specimens were identified to the species level whenever possible, using specialized dichotomous keys and preserved in the Entomological Collection TB, Unical. Various trap types captured a different number of species: the PaT collected 32 species, followed by the PT with 24, the MT with 16, the VC with 7, and the BBT with 5 species. Interestingly, biodiversity analyses conducted using PAST software version 4.17 revealed that PaTs and MTs recorded the highest biodiversity indices. The GLMM analysis, performed using SPSS software 29.0.1.0, demonstrated that various traps attracted different species with different abundances. By combining multiple trapping techniques, we documented a more comprehensive community composition compared to single-method approaches. Moreover, PaTs, MTs, and PTs recorded 20%, 40%, and 33% of the Near Threatened species, respectively. We report new records for Sila National Park, including the LC species *Pteryngium crenulatum* (Curculionidae) and the NT species *Grynocharis oblonga* (Trogossitidae). For the first time in Calabria, the LC species *Triplax rufipes* (Erotylidae) and the NT species *Oxypleurus nodieri* (Cerambycidae) and *Glischrochilus quadrisignatus* (Nitidulidae) were collected. Our results emphasize the importance of method diversity in capturing species with distinct ecological requirements and highlight the relevance of saproxylic beetles as indicators of forest health. These findings support the adoption of multi-method sampling protocols in forest biodiversity monitoring and management programs, especially in biodiversity-rich and structurally heterogeneous landscapes.

## Simple Summary

Saproxylic beetles play a key role in forest ecosystem functioning, particularly in wood decomposition and nutrient cycling. However, their populations are increasingly threatened by human activity and climate change. This study, conducted in the Sila National Park (southern Italy) area, evaluated the effectiveness of integrative sampling methods for monitoring saproxylic beetle diversity. Beetle communities were surveyed in two beech and four pine forests using five complementary sampling techniques: Pan Traps, Malaise Traps, Pitfall Traps, Bait Bottle Traps, and Visual Census. Combining these methods enabled a more comprehensive assessment of community composition than any single method alone. Our findings highlight the importance of multi-method approaches in forest biodiversity monitoring and emphasize their utility for ecosystem health and guiding conservation efforts, particularly in complex, species-rich Mediterranean forests.

## 1. Introduction

Deadwood-associated (saproxylic) beetles represent a key component of forest biodiversity and play vital ecological roles in nutrient cycling, wood decomposition, and trophic interactions [1,2,3]. Due to their sensitivity to microhabitat changes, structural continuity, and deadwood availability, saproxylic beetles are widely regarded as valuable bioindicators for assessing forest habitat quality and ecological integrity [3,4,5,6]. Despite their ecological significance, these beetles are experiencing widespread declines, driven by habitat loss, forest homogenization, intensive management, and the removal of deadwood, an essential substrate for their survival [2,3,5,7,8,9,10,11,12,13,14,15,16,17,18]. In addition, even if in some areas there is a growing awareness of deadwood’s ecological value, there is a severe scarcity of highly decomposed wood. This critical deficit in habitat and resources directly impacts many organisms, including numerous insect species, as their life cycles are entirely dependent on these specific conditions [19]. Mediterranean forest ecosystems, while often underrepresented in saproxylic research, harbor a high degree of biodiversity and structural complexity, particularly in areas with low disturbance histories such as national parks and reserves [4]. In these ecosystems, traditional forest management practices, such as the systematic removal of Coarse Woody Debris, have historically reduced the availability of key habitats for saproxylic species [20]. Monitoring these communities is therefore crucial for understanding forest health and informing sustainable forest management and conservation strategies.

Forest management within protected areas aims to preserve the forest structure by avoiding the removal of dead wood on the ground and by implementing selective tree-cutting practices [1]. Several studies have shown that microhabitats in both living and dead trees are crucial for the survival and reproduction of many insect species [21]. However, to improve our understanding of the relationships between forest management practices, microhabitat availability. and biodiversity, particularly in Mediterranean areas, further research is needed [10]. Regarding the factors affecting the species richness and community composition of saproxylic species, it is well established that characteristics such as the type of dead wood, the position of dead trunks, trunk diameter, wood decay stage [14], and the specific microclimate, particularly sun exposure [3,6,15,16,22,23], play a key role.

Due to their drastic decline, many saproxylic beetles are protected by national and international legislation and are listed on the IUCN Red List. In Central Europe, about 40% of these species are red-listed [16], and in Italy, 2049 beetle species across 66 families are recorded [24]. Effective conservation requires adapting forest management strategies, with protected areas playing a key role by preserving old-growth habitats. Even small forest patches or isolated ancient trees can serve as refuges for rare and relict beetle populations [3,15].

Calabria’s forests, in southern Italy, have experienced extensive deforestation since ancient times, intensified by Roman and agricultural expansion. However, during the 20th century, this trend reversed, leading to an 83% increase in forest area. Today, forests cover approximately 613,000 hectares [25]. Within the Sila landscape, the Sila National Park spans 73,695 hectares and includes several Sites of Community Importance (SICs). It features macrothermic beech forests alongside stands of *Pinus nigra* ssp. *laricio* and supports high insect biodiversity [26,27]. Saproxylic insects are especially important here, serving as bioindicators of forest health and maturity [3,7,10,11,12]. Many saproxylic species are small, cryptic, or associated with specific subcortical habitats, making them difficult to detect [18,28]. For effective sampling and monitoring, a variety of non-invasive methods and traps are essential to obtain representative data [29,30,31,32]. Commonly used techniques include Window Traps for flying insects [17,33,34], Sticky Traps for studying colonization processes [22], Pitfall Traps in tree cavities [35,36], and Elector Traps for insects emerging from dead wood [37]. In addition, important sampling methods include rearing sacks and Tullgren funnels [38], which are particularly effective for collecting species associated with Fine Woody Debris, fungi, and various stages of decaying wood. Cryptic and immature species are often collected manually from under tree bark [39,40,41].

A significant methodological challenge in saproxylic beetle research is the effective sampling of diverse assemblages. Different species exhibit varied life history traits, microhabitat preferences, and dispersal behaviors, which often require the application of complementary sampling techniques (e.g., Flight Interception Traps, Baited Traps, Window Traps) to adequately capture the community structure [33]. Integrated sampling approaches are increasingly recommended to overcome the selectivity and biases associated with single-method studies, particularly in complex habitats with high conservation value.

In this study, we assess the diversity of saproxylic beetles in Sila National Park (Calabria, southern Italy), a protected Mediterranean montane forest landscape characterized by high structural heterogeneity and minimal human disturbance. We evaluate the effectiveness of combined trapping methods for broadly sampling entomofauna, with a focus on saproxylic beetles, to provide a comprehensive assessment of their assemblages. Our findings have international relevance, as developing efficient, broad-spectrum sampling techniques is essential for harmonizing biodiversity monitoring worldwide, informing conservation strategies, and enabling robust comparisons across forest types. This study aims to (i) document saproxylic beetle species’ richness and composition, (ii) compare the performance of different trapping methods, (iii) discuss implications for forest biodiversity monitoring and management, and (iv) evaluate the proportion of species of conservation concern captured by each trap type, comparing our data with those from other studies. Based on the distinct designs and functions of the unconventional traps, we hypothesize that trapping efficiency will vary significantly between trap types, with some being more effective at capturing species of conservation concern and cryptic species, thereby supporting more comprehensive biodiversity assessments and targeted conservation efforts.

## 2. Materials and Methods

### 2.1. Sampling Area

**Figure 1:**
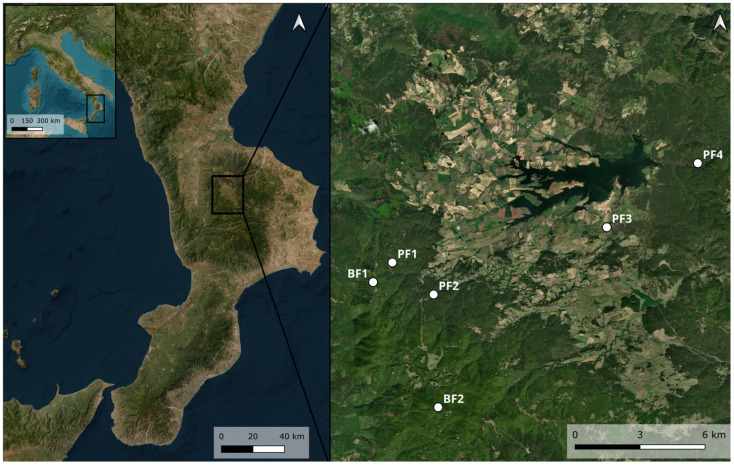
Sampling areas on the Sila plateau, Calabria (southern Italy). On the right are the sampling areas (QGIS Desktop 3.44.1).

Saproxylic insects were collected as part of an entomological study conducted on the Silan Plateau (Calabria, southern Italy). Sampling occurred between 2020 and 2021 for areas PF1, PF2, FB1, and FB2 and between 2022 and 2023 for areas PF3 and PF4. The study sites, sampled from May to November, included two beech forests (BF1 and BF2) and four pine forests (PF1, PF2, PF3, and PF4) (Figure 1).

BF1 (39°20′19.4″ N, 16°23′50.3″ E) is located at 1580 m above sea level in the locality of Vette (Moccone municipality). The forest is predominantly composed of *Fagus sylvatica* L., with occasional specimens of *Abies alba* Mill. BF2 (39°17′13.0″ N, 16°25′55.8″ E) lies at 1820 m above sea level and is accessible only via the Vette area. It consists mainly of *Fagus sylvatica*, with a few scattered *Abies alba* trees. PF1 (39°20′48.6″ N, 16°24′27.5″ E) is situated at 1425 m above sea level near the locality of Fago del Soldato (Celico municipality). This forest is dominated by *Pinus nigra*, with a few *Fagus sylvatica* individuals. The understory includes *Erica arborea* L. and *Spartium junceum* L. and features numerous Standing Dead Trees and a significant amount of Fine Woody Debris on the forest floor. PF2 (39°20′01.0″ N, 16°25′47.0″ E) is located at 1320 m above sea level in the Camigliatello Silano municipality. This pine forest is dominated by *Pinus nigra*, with the presence of some *Fagus sylvatica* L. and *Populus alba* L. individuals.

The understory is composed mainly of *Rubus* spp. and *Pteridium aquilinum* (L.), along with scattered Fine Woody Debris on the forest floor.

PF3 (39°21′41.0″ N, 16°31′21.0″ E), situated at 1192 m above sea level in the Spezzano della Sila municipality, is a pine forest bordering an agricultural potato field. It consists primarily of *Pinus nigra* and features a grassy understory. The area is subject to seasonal grazing by livestock, particularly from mid-August through the autumn.

**Figure 2:**
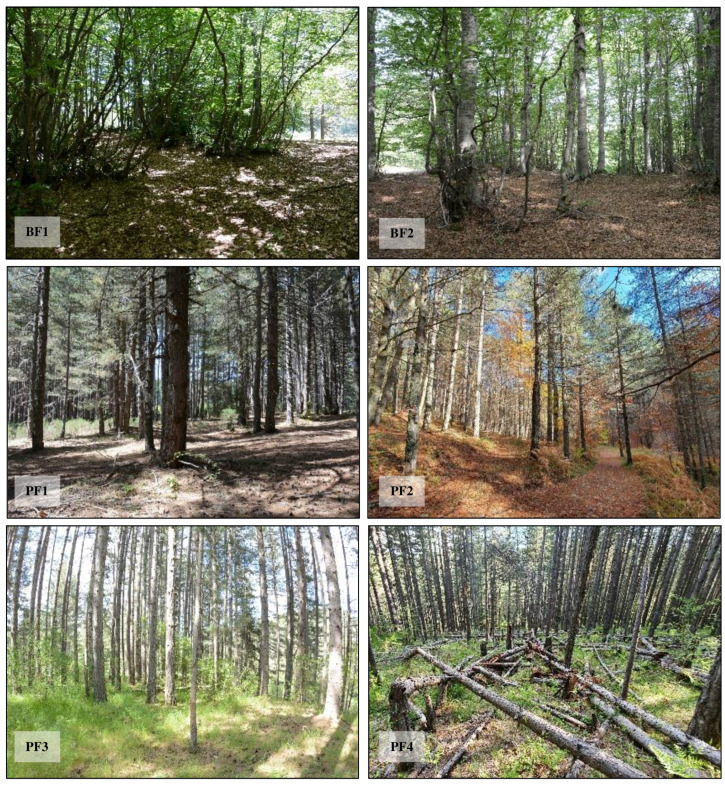
Sampling sites for saproxylic insect collection. BF1: Beech Forest 1; BF2: Beech Forest 2; PF1: Pine Forest 1; PF2: Pine Forest 2; PF3: Pine Forest 3; PF4: Pine Forest 4.

PF4 (39°23′16.3″ N, 16°34′16.5″ E), at 1275 m above sea level in the Longobucco municipality, is a *Pinus nigra*-dominated forest characterized by the presence of numerous decaying fallen trunks. The understory includes *Pteridium aquilinum*, *Epipactis* spp., *Rubus* spp., and *Fragaria vesca* L. (Figure 2).

Site quality was assessed based on the following criteria: (i) forest management practices, (ii) presence of necromass, and (iii) tree age [41,42]. Low-quality forests were characterized by overexploitation, removal of necromass, and the presence of recent plantations. In contrast, high-quality forests exhibited a high degree of naturalness, abundant woody necromass, and the presence of old-growth trees (Table 1). Following previous classifications [7,43], necromass was categorized into Standing Dead Trees (SDTs), Coarse Woody Debris (CWD), and Fine Woody Debris (FWD). In this study, woody necromass was surveyed within a 200 × 200 m plot, with the diameter of fallen dead trees also recorded.

### 2.2. Insects’ Collection

Insect sampling was carried out using Pan Traps, Malaise Traps, Pitfall Traps, and Bait Bottle Traps. A total of one Pan Trap was placed at each site. Each Pan Trap consisted of three containers of different colors (blue, white, and yellow), each with a diameter of 15 cm and a depth of 25 cm. The traps were positioned 50 cm above the ground and secured using wooden stakes. They were filled with water mixed with odorless soap and left in the field for 15 days [44].

At each site, a Malaise Trap was placed at a distance of 30 m from the other trap types, arranged so that the capture area of one trap did not interfere with another. The arthropod collection container of the Malaise Trap was filled with 70% ethanol [45].

A total of five Pitfall Traps were placed at each site. Each trap consisted of a plastic cup (diameter: 8.5 cm, height: 12 cm) filled to one-third with a saturated saline solution of white vinegar and a few drops of detergent to kill and preserve the captured arthropods. The Pitfall Traps were arranged randomly, at least 10 m apart, and placed level with the soil surface [46].

At each site, five bait bottle traps were installed. The Bait Bottle Traps (volume: 2 L) were set up as previously described [47]. Inside the lower chamber, two plastic containers were placed: a 50 mL container with 25 g of bovine liver and 20 mL of saturated NaCl solution, and a 100 mL container with 50 g of bovine liver and 40 mL of liquid protein bait (Dacus trap^®^, BioIberica, Barcelona, Spain). Distances between Bait Bottle Traps ranged from 25 to 30 m. The traps were attached to tree trunks or poles at a height of 1.70–1.80 m using cable ties [48].

**Figure 3:**
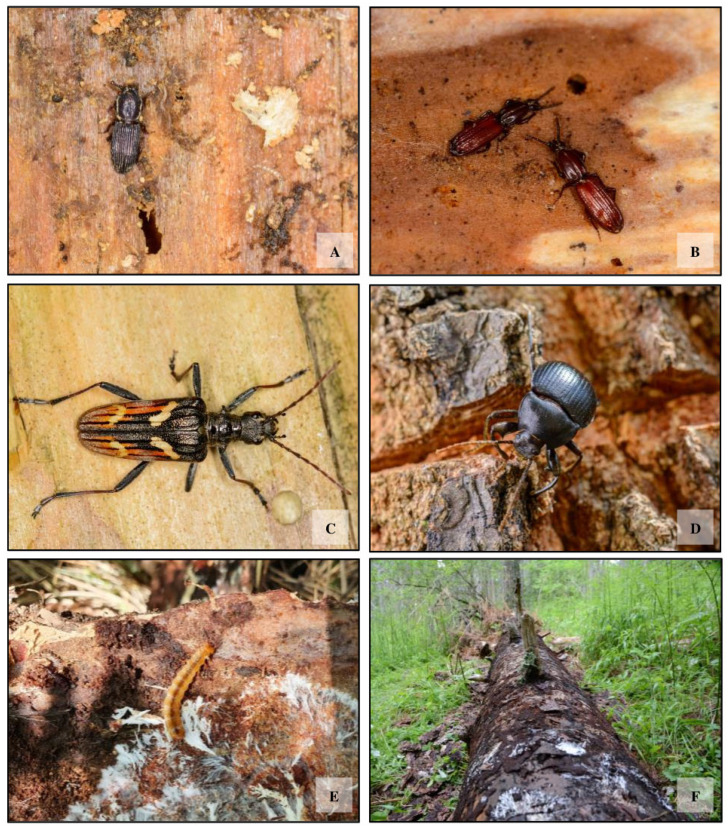
Photos of selected saproxylic beetles. (**A**) *Pycnomerus italicus*, (**B**) *Clinidium canaliculatum*, (**C**) *Rhagium bifasciatum*, (**D**) *Enoplopus dentipes*, (**E**) larva of *Cucujus cinnaberinus* and (**F**) woody necromass.

In addition, insects were collected by hand from inside decaying trunks, using aspirators, vacuum cleaners, and tweezers (Figure 3). Trap samples were collected every 15 days, then examined and identified at the General and Applied Entomology Laboratory of the University of Calabria. All specimens were identified using specialized dichotomous keys [49,50,51,52], preserved in 60% alcohol, and left to dry in the Entomological Collection TB of DiBEST, Unical.

### 2.3. Data Analysis

The data were statistically analyzed to calculate biodiversity indices (Shannon, Evenness, Margalef, and Simpson) and to perform the Kruskal–Wallis test. These analyses were conducted using PAST software version 4.17. The relationship graph between species and sampling methods was drawn using SPSS software version 29.0.1.0. A Generalized Linear Mixed Model (GLMM) [53], performed by SPSS software version 29.0.1.0, was employed to evaluate the effects of the trap type and species on insect abundance, with the site and year included as random effects to account for the hierarchical structure of the data. The model assumed a negative binomial distribution for the dependent variable (abundance) and used a logarithmic link function.

## 3. Results

Our sampling yielded 544 collected and identified specimens, comprising 63 species across 23 distinct families. The most species-rich families were Cerambycidae, Tenebrionidae, and Nitidulidae. A complete and detailed checklist of all identified species, including sampling details and their conservation statuses, is provided in the Supplementary Material (Table S1).

Bait Bottle Traps (BBTs) were particularly effective at attracting species sensitive to olfactory stimuli, while Pitfall Traps (PTs) were more efficient for ground-dwelling species or those associated with microhabitats such as litter and soil organic matter. Malaise Traps were especially effective for flying insects, consistent with the existing literature. Data analysis (Table 2) confirmed that combining different sampling techniques significantly improves insect collection success. In particular, integrating the Visual Census (VC) method with trap-based approaches helps compensate for the limitations of each individual method. This strategy reduces biases related to insect behavior, environmental variability, and observer influence. For example, BBTs may overestimate the abundance of species attracted to specific chemical cues, while a VC tends to underestimate small or cryptic species.

**Figure 4:**
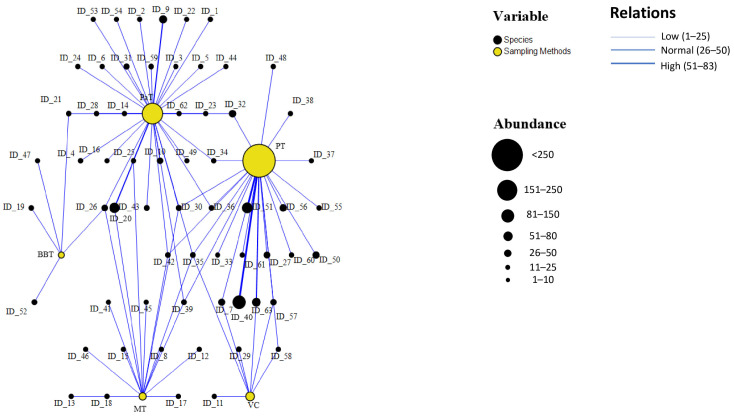
In the relationship graph, interactions between the different sampling traps (represented by yellow nodes) and the captured species (black nodes) are visualized. The size of each node is proportional to the total number of captures, while each line (edge) connects a trap to a species. The thickness of the line indicates the strength of the association, i.e., the abundance of captures made by a given trap. The traps include Bait Bottle Traps (BTs), Pan Traps (PaTs), Malaise Traps (MTs), Pitfall Traps (PTs), and Visual Census (VC). The graph was generated using SPSS software version 29.0.1.0.

In the graph (Figure 4), interactions between different sampling traps (represented by yellow nodes) and the captured species (black nodes) are visualized. The primary aim of this analysis was to evaluate the efficiency of each trap, specifically identifying which methods are most effective in terms of both specimen abundance and species diversity. The sampling methods that captured the highest number of individuals were Pitfall Traps (PTs), Pan Traps (PaTs), and the Visual Census (VC). A high number of connecting lines between a single trap and multiple species indicates greater versatility, meaning the trap is effective at capturing both numerous individuals and a diverse range of species.

Some species were captured by multiple trap types, such as *Pediacus dermestoides* (ID_30), a Near Threatened (NT) species, collected by PTs, VC, PaTs, and Malaise Traps (MTs); *Thanasimus formicarius* (ID_26), a Least Concern (LC) species, caught by PTs, MTs, and Bait Bottle Traps (BBTs); and *Ampedus sinuatus* (ID_35), a Vulnerable (VU) species, sampled using PTs, VC, PaTs, and MTs. Other species were captured exclusively by a single trap type, including *Anastrangalia dubia* (ID_9, LC), collected only with PTs; *Mycetophagus quadripustulatus* (ID_47, LC), collected only with BBTs; *Dacne rufifrons* (ID_38, NT), captured only with PaTs; *Pogonocherus decoratus* (ID_15, NT), collected only with MTs; and *Cucujus cinnaberinus* (ID_29, VU), captured exclusively by VC.

Figure 4 highlights that Pitfall Traps (PTs) and Pan Traps (PaTs) are particularly effective for sampling large numbers of species, especially floral visitors with high dispersal ability, such as the macropterous *Anastrangalia sanguinolenta* (ID_10) [54]. In contrast, species with limited dispersal capacity and those associated with litter such as certain Cryptophagidae like *Pteryngium crenulatum* (ID_27) [55] are more frequently collected using PTs. Some ground-dwelling species collected by PTs were also recorded by the Visual Census, while several flying species captured with PaTs were also sampled using Malaise Traps (MTs).

Among the species classified in IUCN risk categories, only species of Least Concern (LC), such as *Rhagium inquisitor* (ID_19), were collected using Bait Bottle Traps (BBTs). In contrast, Pan Traps (PaTs), Malaise Traps (MTs), and Pitfall Traps (PTs) showed a more heterogeneous distribution across risk categories. While LC species still constitute a large proportion of the captures, notable percentages of species classified as Vulnerable (VU) were also recorded 7% for both PaTs and MTs, and 8% for PTs. More significantly, species categorized as Near Threatened (NT) represented 20% of captures in PaTs, 40% in MTs, and 33% in PTs. These results indicate that PaTs, MTs, and PTs are more effective in capturing a broader range of taxa, including potentially more Vulnerable species. Specifically, Pitfall Traps were more efficient at sampling species associated with soil or litter habitats (e.g., *Triplax marseuli*, ID_40), whereas Malaise Traps predominantly captured flying insects (e.g., *Purpuricenus globulicollis*, ID_17). The Endangered species *Pycnomerus italicus* was primarily collected using Pitfall Traps (27 individuals) and the Visual Census (10 individuals), suggesting that these methods may be especially effective for detecting cryptic or small-bodied taxa.

Finally, the Visual Census (VC) mainly yielded LC species, but also included VU and NT species, although in lower numbers and diversity compared to PaTs, PTs, and MTs.

Within the collected species (Table S1, Supplementary Material), we report new records for Sila National Park, including the LC species *Pteryngium crenulatum* (Curculionidae), collected in PF2 via PTs, and the NT species *Grynocharis oblonga* (Trogossitidae), collected in PF4 via PTs. For the first time in Calabria, the LC species *Triplax rufipes* (Erotylidae) was recorded in PF3 and PF4 via MTs, and the NT species *Oxypleurus nodieri* (Cerambycidae) was collected in PF3 via MTs, as well as *Glischrochilus quadrisignatus* (Nitidulidae) in PF3 and PF4 via PTs. Our data show that using multiple trap types for insect sampling is highly recommended to obtain a more comprehensive faunistic dataset, especially when the research goal is to study rare or high-risk insect species. Employing several sampling methods increases the likelihood of detecting a greater number of species, which is crucial for accurately assessing the conservation status of target communities and developing effective management strategies.

The analysis of fixed effects revealed that the overall model was highly significant (F(92,36) = 2.758, *p* < 0.001), indicating that the predictor variables explained a significant portion of the variation in specimen abundance. Specifically, the trap type had a statistically significant effect (F(4,36) = 3.468, *p* = 0.017). The PaT had the highest estimated mean abundance (2.891), followed by PTs (2.444), MTs (1.685), VC (0.850), and BBTs (0.500). While these estimates suggest a relative ranking in trap efficiency, pairwise comparisons between specific traps were not conducted in this analysis.

Species identity also had a highly significant effect on abundance (F(88,36) = 2.158, *p* = 0.006), confirming substantial natural variation among species in the number of individuals captured. The estimated variances for the random effects were examined to assess variability across sites and years. The variance attributed to the site was very small and not statistically significant (Variance = 0.002, SE = 0.055, Z = 0.040, *p* = 0.968). Similarly, the variance for the year was estimated as zero (Variance = 0.000), and its standard error and *p*-value could not be calculated, as the estimate reached the boundary of its parameter space.

The negligible and non-significant variances for both the site and year suggest that the baseline abundance did not vary meaningfully across spatial or temporal dimensions in this study. This reinforces the conclusion that the significant effects of fixed factors, particularly the trap type, are consistent and generalizable across the sampled sites and years.

Despite some boundary estimates in the random effects, the model yielded interpretable and meaningful fixed-effect results, supporting this study’s main research hypotheses.

## 4. Discussion

Saproxylic beetles are key components of forest ecosystems, contributing to nutrient cycling, wood decomposition, and trophic interactions. Their strong dependence on deadwood and other decaying substrates makes them highly sensitive to changes in forest structure and management, positioning them as reliable bioindicators of habitat quality and ecological continuity. However, this group is undergoing a pronounced decline due to anthropogenic pressures such as habitat fragmentation, deadwood removal, pollution, and climate change. In this context, continuous and standardized monitoring of saproxylic beetle assemblages is essential for informing evidence-based forest management and conservation strategies. Evaluating the diversity and composition of these assemblages offers valuable insights into ecosystem integrity and the effects of silvicultural practices. Moreover, saproxylic beetles represent a useful model system for studying ecological and evolutionary processes due to their high species richness, wide range of ecological niches, and frequent occurrence of cryptic or poorly known taxa. The results of this study emphasize the importance of employing a multi-method sampling strategy to effectively assess saproxylic beetle diversity. The integration of different trap types—including Bait Bottle Traps, Pan Traps, Malaise Traps, Pitfall Traps, and Visual Surveys—allowed the detection of species with differing ecological traits and microhabitat preferences. This approach proved more effective than single-method surveys, which risk underestimating biodiversity and overlooking key components of the saproxylic community.

The value of integrated sampling approaches for assessing saproxylic beetle communities has been demonstrated in previous studies. For instance, Ranius and Jansson [35] compared Window Traps and Pitfall Traps, showing that while Window Traps captured a greater number of flying species, Pitfall Traps were more selective for species inhabiting or moving within wood cavities. Their findings emphasized the importance of combining methods to effectively sample different components of the saproxylic assemblage. This principle of methodological complementarity was also evident in our study in Sila National Park. By employing a combination of Pan Traps, Malaise Traps, Pitfall Traps, Bait Bottle Traps, and Visual Census, we were able to overcome the limitations of individual methods and achieve a more comprehensive and representative assessment of saproxylic beetle diversity.

Similarly, Redolfi De Zan et al. [56] surveyed saproxylic beetles in three relict beech forests in central Italy using Pitfall Traps and various types of Window Traps. Their study, in line with our findings, highlighted the influence of the trap type on community composition: Pitfall Traps were particularly effective for species associated with soil or cavities, while Flight Interception Traps targeted flying taxa.

Our results demonstrate that sampling effectiveness is significantly influenced by the type of trap employed. The integration of five complementary methods, including less commonly used techniques such as Pan Traps, enabled the collection of a broader spectrum of taxonomic and functional diversity. Notably, this multi-method approach was essential for detecting species of high ecological and conservation value, leading to a more accurate estimation of saproxylic community composition, including rare and cryptic species. These findings support our initial hypothesis and highlight the importance of employing methodologically diverse strategies in monitoring studies conducted in forest environments characterized by high structural complexity.

Given the conservation relevance of forest ecosystems such as those in Sila National Park, the adoption of integrative and long-term monitoring protocols is strongly recommended. Such frameworks can support adaptive management by identifying priority habitats, evaluating the effectiveness of conservation measures, and guiding the retention of key structural elements like deadwood and senescent trees. In conclusion, sustained investment in saproxylic beetle monitoring, using complementary sampling techniques, is crucial for both biodiversity conservation and the development of ecologically informed forest management. This study highlights the role of saproxylic beetles not only as indicators of ecosystem health but also as focal taxa for enhancing forest resilience in the face of environmental change.

## 5. Conclusions

Our study highlights the crucial role of methodological diversity in reducing sampling bias and improving biodiversity detection, particularly in understudied Mediterranean forest ecosystems. By providing a more comprehensive assessment of saproxylic beetle communities and their ecological associations, our findings contribute to the refinement of forest biodiversity monitoring protocols and expand upon previous research. To our knowledge, no prior studies have explicitly evaluated the effectiveness of Pan Traps for sampling saproxylic beetles, making this study a novel contribution. We tested, for the first time, the use of Pan Traps as a complementary method alongside more conventional approaches such as Pitfall Traps and Visual Census. The results demonstrate that Pan Traps can effectively capture a substantial number of species, including several of conservation concern. This effectiveness likely stems from the floral mimicry of Pan Traps, which attract adult beetles that also act as flower visitors. Their color and shape simulate floral resources, making them appealing to species that are often underrepresented in traditional sampling. Incorporating Pan Traps into multi-method sampling frameworks expands the detectable taxonomic and functional diversity and provides new opportunities for monitoring cryptic or rarely encountered species. Overall, our findings support the integration of complementary sampling methods including less commonly used techniques as essential for comprehensive biodiversity assessments and the development of targeted conservation strategies. Future research should further investigate the floral preferences and behavioral ecology of saproxylic beetles in relation to trap design, with the goal of optimizing Pan Trap performance across different forest contexts.

## Figures and Tables

**Table 1:** Characteristics of sampling sites: site code, altitude (m a.s.l.), site quality, number of Standing Dead Trees (SDTs) and average diameter (cm) and standard deviation (SD), number of Coarse Woody Debris (CWD) and average diameter (cm) and SD, number of Fine Woody Debris (FWD) and average diameter (cm) and SD, and dominant tree species. HQ, high-quality forest biotope; MQ, medium-quality forest biotope; and LQ, low-quality forest biotope.

Site Code	Altitude (in Meters a.s.l.)	Site Quality	SDT	CWD	FWD	Tree Species
BF1	1580 m	MQ	-	-	30 (4.010 ± 1.30)	*F. sylvatica*
BF2	1820 m	HQ	-	-	71 (4.26 ± 1.15)	*F. sylvatica*
PF1	1425 m	MQ	24 (15.54 ± 2.51)	-	84 (5.30 ± 2.03)	*P. nigra*
PF2	1320 m	MQ	-	-	18 (3.72 ± 1.18)	*P. nigra* and *F. sylvatica*
PF3	1192 m	MQ	4 (17.25 ± 5.97)	-	10 (7 ± 2.45)	*P. nigra*
PF4	1275 m	HQ	46 (27.30 ± 10.88)	50 (34.58 ± 9.55)	14 (7.64 ± 2.34)	*P. nigra*

**Table 2:** Summary table of sampling results using different capture methods: Bait Bottle Traps (BBTs), Pan Traps (PaTs), Malaise Traps (MTs), Pitfall Traps (PTs), and Visual Census (VC). The table presents the number of species and individuals collected, the standard deviation, and biodiversity indices (Shannon, Evenness, Simpson, and Margalef) that describe the diversity and evenness of the communities sampled by each method.

	BBT	PaT	MT	PT	VC
Species	5	32	16	24	7
Specimens	17	166	30	302	30
SD	1.1801	7.469	1.268	13.536	1.740
Dominance	0.272	0.136	0.0965	0.138	0.198
Simpson	0.728	0.864	0.903	0.862	0.802
Shannon	1.452	2.674	2.715	2.386	1.743
Evenness	0.854	0.453	0.944	0.453	0.816
Margalef	1.412	6.064	4.41	4.028	1.764

## Data Availability

The raw data supporting the conclusions of this article will be made available by the authors upon request.

## Supplementary Materials

The following supporting information can be downloaded at https://www.mdpi.com/article/10.3390/insects16080812/s1, Table S1: Checklist of saproxylic species collected.

## References

[1] Grove S.J. (2002). Saproxylic insect ecology and the sustainable management of forests. Annu. Rev. Ecol. Evol. Syst..

[2] Lassauce A., Paillet Y., Jactel H., Bouget C. (2011). Deadwood as a surrogate for forest biodiversity: Meta-analysis of correlations between deadwood volume and species richness of saproxylic organisms. Ecol. Indic..

[3] Tsikas A., Karanikola P. (2022). To Conserve or to Control? Endangered Saproxylic Beetles Considered as Forest Pests. Forests.

[4] Nieto A., Alexander K.N.A. (2010). European Red List of Saproxylic Beetles.

[5] Calix M., Alexander K.N.A., Nieto A., Dodelin B., Soldati F., Telnov D., Albalate Z.V., Aleksandrowicz O., Audisio P., Istrato P. (2018). European Red List of Saproxylic Beetles.

[6] Parisi F., Mazziotta A., Vangi E., Tognetti R., Travaglini D., Marchetti M., D’Amico G., Francini S., Borghi C., Chirici G. (2023). Exposure elevation and forest structure predict the abundance of saproxylic beetles’ communities in mountain managed beech forests. iForest.

[7] Audisio P., Baviera C., Carpaneto G.M., Biscaccianti A.B., Battistoni A., Teofili C., Rondinini C. (2014). Lista Rossa IUCN dei Coleotteri Saproxilici Italiani.

[8] Seibold S., Bässler C., Brandl R., Gossner M.M., Thorn S., Ulyshen M., Müller J. (2015). Experimental studies of dead-wood biodiversity. A review identifying global gaps in knowledge. Biol. Conserv..

[9] Hardersen S., Zapponi L. (2018). Wood degradation and the role of saproxylic insects for lignoforms. Appl. Soil Ecol..

[10] Parisi F., Lombardi F., Marziliano P.A., Russo D., De Cristofaro A., Marchetti M., Tognetti R. (2020). Diversity of saproxylic beetle communities in chestnut agroforestry systems. iForest.

[11] Zumr V., Remes J., Pulkrab K. (2021). How to Increase Biodiversity of Saproxylic Beetles in Commercial Stands through Integrated Forest Management in Central Europe. Forests.

[12] Farashiani M.E., Varandi H.B., Kazerani F., Yarmand H., Babaee M., Thorn S., Lange F., Rafiei-Jahed R., Müller J., Amini S. (2022). A preliminary checklist of saproxylic beetles (Coleoptera) in the Hyrcanian forests of Iran, with distributional data. Check List.

[13] Winiger N., Handel A.L., Ganz S., Zielewska-Buttner K., Segelbacher G., Braunisch V. (2023). Saproxylic beetles respond to habitat variables at different spatial scales depending on variable type and species’ mobility: The need for multi-scale forest structure management. Biodivers. Conserv..

[14] Vogel S., Bussler H., Finnberg S., Müller J., Stengel E., Thorn S. (2021). Diversity and conservation of saproxylic beetles in 42 European tree species: An experimental approach using early successional stages of branches. Insect Conserv. Divers..

[15] Carpaneto G.M., Baviera C., Biscaccianti A.B., Brandmayr P., Mazzei A., Mason F., Battistoni A., Teofili C., Rondinini C., Fattorini S. (2015). A Red List of Italian saproxylic beetles: Taxonomic overview, ecological features and conservation issues (Coleoptera). Fragm. Entomol..

[16] Zumr V., Nakladal O., Gallo J., Remes J. (2024). Deadwood position matters: Diversity and biomass of saproxylic beetles in a temperate beech forest. For. Ecosyst..

[17] Campanaro A., Parisi F. (2021). Open datasets wanted for tracking the insect decline: Let’s start from saproxylic beetles. Biodivers. Data J..

[18] Parisi F., Vangi E., Francini S., Chirici G., Travaglini D., Marchetti M., Tognetti R. (2022). Monitoring the Abundance of Saproxylic Red-Listed Species in a Managed Beech Forest by Landsat Temporal Metrics. For. Ecosyst..

[19] Motta R. (2020). Perché dobbiamo aumentare la quantità di necromassa nelle nostre foreste? Quanta necromassa nelle dobbiamo rilasciare?. Forest.

[20] Buse J., Ranius T., Assmann T. (2008). An endangered longhorn beetle associated with old oaksand its possible role as an ecosystem engineer. Conserv. Biol..

[21] Bujoczek L., Bujoczek M. (2022). Factors influencing the diversity of deadwood, a crucial microhabitat for many rare and endangered saproxylic organisms. Ecol. Indic..

[22] Graf M., Lettenmaier L., Muller J., Hagge J. (2021). Saproxylic beetles trace deadwood and differentiate between deadwood niches before their arrival on potential hosts. Insect Conserv. Divers..

[23] Hagge J., Muller J., Bassler C., Brandl R., Schuldt A., Thorn S., Seibold S. (2024). Change in saproxylic beetle, fungi and bacteria assemblages along horizontal and vertical gradients of sun-exposure in forest. Biol. Conserv..

[24] Amori G., Mazzei A., Storino P., Urso S., Luzzi G., Aloise G., Gangale C., Ouzounov D., Luiselli L., Pizzolotto R. (2022). Forest management and conservation of faunal diversity in Italy: A review. Plant Biosyst..

[25] Iovino F., Nicolaci A. (2016). Disboscamenti in Calabria: Cause storiche, conseguenze e rimedi. L’Italia For. E Mont..

[26] Brandmayr P., Gangale C., Mazzei A., Mingozzi A., Pizzolotto R., Urso S., Scalercio S., Tripepi S., Aloise G., Ouzunov D. (2013). L’approfondimento: La Biodiversità Animale e Vegetale Della Sila. Sinergie Rapp. Di Ric..

[27] Mazzei A., Brandmayr P., Contarini E., Luzzi G. (2016). I Coleotteri Del Parco Nazionale della Sila. Specie Saproxilobionti Di Maggior Interesse Comunitario, Faunistico E Conservazionistico.

[28] Audisio P., Mancini E., Sabatelli S. (2016). Unraveling cryptic species diversity in an aposematic sap beetle genus (Coleoptera: Nitidulidae: Cryptarchinae) from northern Europe. Insect Syst. Evol..

[29] Honek A. (1988). The effect of crop density and microclimate on pitfall trap catches of Carabidae, Staphyiinidae (Coleóptera), and Lycosidae (Araneae) in cereal fields. Pedobiologia.

[30] Brandmayr P., Zetto T., Pizzolotto R. (2005). I Coleotteri Carabidi Per La Valutazione Ambientale E La Conservazione Della Biodiversità. Manuale Operativo.

[31] Greco S., Brandmayr P., Bonacci T. (2014). Synanthropy and temporal variability of Calliphoridae living in Cosenza (Calabria, Southern Italy). J. Insect Sci..

[32] Montgomery G.A., Belitz M.W., Guralnick R.P., Tingley M.W. (2021). Standards and Best Practices for Monitoring and Benchmarking Insects. Front. Ecol. Evol..

[33] Bouget C., Brustel H., Brin A., Noblecourt T. (2008). Sampling Saproxylic Beetles with Window Flight Traps: Methodological Insights. Rev. Écol Terre Vie.

[34] Martikainen P., Kaila L. (2004). Sampling saproxylic beetles: Lessons from a 10-year monitoring study. Biol. Conserv..

[35] Ranius T., Jansson N. (2002). A comparison of three methods to survey saproxylic beetles in hollow oaks. Biodivers. Conserv..

[36] Vrezec A., Ambrožič Š., Kapla A. (2012). An Overview of Sampling Methods Tests for Monitoring Schemes of Saproxylic Beetles in the Scope of Natura 2000 in Slovenia. Stud. For. Slov..

[37] Alinvi O., Ball J.P., Danell K., Hjältén J., Pettersson R.B. (2007). Sampling saproxylic beetle assemblages in dead wood logs: Comparing window and eclector traps to traditional bark sieving and a refinement. J. Insect Conserv..

[38] Jonsell M., Hansson J. (2007). Comparison of methods for sampling saproxylic beetles in fine wood. Entomol. Fenn..

[39] Bonacci T., Mazzei A., Horak J., Brandmayr P. (2012). *Cucujus tulliae* sp., an endemic Mediterranean saproxylic beetle from genus *Cucujus* Fabricius, 1775 (Coleoptera, Cucujidae), and keys for identification of adults and larvae native to Europe. ZooKeys.

[40] Bonacci T., Biscaccianti A.B., Siclari A., Carlomagno F., Bonelli D., Mendicino F., Plewa R., Jaworski T., Pezzi M. (2022). Presence of the endangered saproxylic species *Cucujus haematodes* (Coleoptera: Cucujidae) in Aspromonte National Park (Southern Italy). Eur. Zool. J..

[41] Pezzi M., Carlomagno F., Mendicino F., Bonelli D., Pelle R., Leis M., Chicca M., Bonacci T. (2022). *Pycnomerus italicus* (Coleoptera: Zopheridae), an Endemic Endangered Species: A New Report on Its Presence in Southern Italy. Forests.

[42] Mazzei A., Bonacci T., Contarini E., Zetto T., Brandmayr P. (2011). Rediscovering the “umbrella species” candidate *Cucujus cinnaberinus* (Scopoli, 1763) in southern Italy (Coleoptera Cucujidae), and notes on bionomy. Ital. J. Zool..

[43] Morelli S., Paletto A., Tosi V. (2007). Il legno morto dei boschi: Indagine sulla densità basale del legno di alcune specie del Trentino. Forest.

[44] Chinga J., Murùa M., Barahona-Segovia R.M., Gelcich S. (2024). Pan traps: An effective tool for monitoring phenological changes in insect floral visitors and their relationship with floral resources in a coastal Mediterranean forest. Ecol. Indic..

[45] Skvarla M.J., Larson J.L., Fisher J.R., Dowling A.P.G. (2021). A Review of Terrestrial and Canopy Malaise Traps. Ann. Entomol. Soc. Am..

[46] Siewers J., Schirmel J., Buchholz S. (2014). The efficiency of pitfall traps as a method of sampling epigeal arthropods in litter rich forest habitats. Eur. J. Entomol..

[47] Hwang C., Turner B.D. (2005). Spatial and temporal variability of necrophagous Diptera from urban to rural areas. Med. Vet. Entomol..

[48] Pezzi M., Bonelli D., Mendicino F., Carlomagno F., Munari C., Mistri M., Chicca M., Szpila K., Bonacci T. (2024). *Calliphora rohdendorfi* (Grunin, 1966) (Diptera: Calliphoridae): A new blow fly in the Italian fauna detected in Calabrian Apennines. Eur. Zool. J..

[49] Porta A. (1923). Fauna Coleopterorum Italica. Vol I—Adephaga.

[50] Porta A. (1926). Fauna Coleopterorum Italica. Vol II—Staphylinoidea.

[51] Porta A. (1929). Fauna Coleopterorum Italica. Vol III—Diversicornia.

[52] Käfer Europas, Die Käfer Europas Ein Bestimmungswerk im Internet, Herausgegeben von Arved Lompe, Nienburg/Weser, Begründet im September 2002. http://coleonet.de/coleo/index.htm.

[53] Candy S.G. (2000). The application of generalized linear mixed models to multi-level sampling for insect population monitoring. Environ. Ecol. Stat..

[54] Doychev D., Georgiev G. (2004). New and rare longhorn beetles (Coleoptera: Cerambycidae) in Bulgaria. Acta Zool. Bulg..

[55] Pelletier G., Hébert C. (2019). The Cryptophagidae of Canada and the northern United States of America. Can. J. Arthropod Identif..

[56] Redolfi De Zan L., Bellotti F., D’Amato D., Carpaneto G.M. (2014). Saproxylic beetles in three relict beech forests of central Italy: Analysis of environmental parameters and implications for forest management. For. Ecol. Manag..

