# Supplementary material for: Integrated Sampling Approaches Enhance Assessment of Saproxylic Beetle Biodiversity in a Mediterranean Forest Ecosystem (Sila National Park, Italy)
Source: Insects. 2025 Aug 6;16(8):812. doi: 10.3390/insects16080812 (PMC12386947; doi:10.3390/insects16080812)
Supplement: Supplementary file 1 [file insects-16-00812-s001.zip › insects-3775886-supplementary.pdf]

Table S1. Checklist of saproxylic species collected in the sampling areas with their respective trophic categories [7] and Italian [7] and European [4] risk categories. Xy (Xylophagous), Z (Zoophagous), My (Mycophagous), SF (sap-feeder on trees attacked by Xy), Sx (Saproxylophagous on dead wood) [7]. Bait bottle Traps (BBT), Pan Traps (PaT), Malaise Trap (MT), Pitfall Traps (PT), and Visual Census (VC).

ID	Species	IUCN	IUCN Eu	Trophic Categories	PF1	PF2	PF3	PF4	FB1	FB2
				**Buprestidae**						
ID_3	*Acmaeoderella flavofasciata* (Piller & Mitterpacher, 1763)	LC		Xy	0	0	1(PaT)	1(PaT)	0	0
ID_4	*Anthaxia (Melanthaxia) helvetica* Stierlin, 1868	LC		Xy	0	0	1(PaT)	3(PaT)	0	0
ID_5	*Anthaxia (Anthaxia) semicuprea* Kuster, 1851	LC		Xy	0	0	0	1 (PaT)	0	0
ID_6	*Chalcophora mariana* (Linnaeus, 1758)	LC		Xy	0	0	1(PaT)	0	0	0
				**Carabidae**						
ID_7	*Clinidium (Arctoclinidium) canaliculatum* (O.G. Costa, 1839)	NT		Z	0	0	0	24(19PT) (5VC)	0	0
				**Cerambycidae**						
ID_8	*Anaglyptus mysticus* (Linnaeus, 1758)	LC		Xy	0	0	0	0	0	1(MT)
ID_9	*Anastrangalia dubia* (Scopoli, 1763)	LC		Xy	0	0	2(PaT)	28(PaT)	0	0
ID_10	*Anastrangalia sanguinolenta* (Linnaeus, 1761)	LC		Xy	0	0	0	14(PaT)	0	0
ID_11	*Arhopalus rusticus* (Linnaeus, 1758)	LC		Xy	0	0	1(VC)	0	0	0
ID_12	*Deilus fugax* (Olivier, 1790)	LC	LC	Xy	0	0	1(MT)	0	0	0
ID_13	*Oxypleurus nodieri* (Mulsant, 1839)	NT		Xy	0	0	1(MT)	0	0	0
ID_14	*Paracorymbia fulva* (De Geer, 1775)	LC		Xy	0	0	1(PaT)	0	0	0
ID_15	*Pogonocherus decoratus* Fairmaire, 1855	NT		Xy	0	1(MT)	0	1(MT)	0	0
ID_16	*Pseudovadonia livida* (Fabricius, 1776)	LC		Xy	0	0	1(PaT)	0	0	0
ID_17	*Purpuricenus globulicollis* Dejean, 1839	NT		Xy	0	1(MT)	0	0	0	0
ID_18	*Rhagium (Hagrium) bifasciatum* Fabricius, 1775	LC		Xy	0	1(MT)	0	0	0	1(MT)
ID_19	*Rhagium (Rhagium) inquisitor* Linnaeus, 1758	LC		Xy	1(BBT)	3(BBT)	0	0	0	0
ID_20	*Rutpela maculata* (Poda, 1761)	LC		Xy	0	0	1(M)	50(PaT)	1(M)	0
ID_21	*Stenurella bifasciata* ssp. *bifasciata* (O. F. Muller, 1776)	LC		Xy	0	1(BBT)	2(PaT)	2(PaT)	0	0
**ID**	**Species**	**IUCN**	**IUCN Eu**	**Trophic Categories**	**PF1**	**PF2**	**PF3**	**PF4**	**FB1**	**FB2**
				**Cetoniidae**						
ID_22	*Cetonia aurata* subsp. *pisana* Heer, 1841	LC		Xy	0	0	1(PaT)	0	0	0

ID_23	*Valgus hemipterus* Linnaeus, 1758	LC	LC	Xy	0	0	1(PaT)	0	0	0
				**Cleridae**						
ID_24	*Korynetes caeruleus* (De Geer, 1775)	NT		Z	0	0	0	0	0	2(PaT)
ID_25	*Opilo mollis* (Linnaeus, 1758)	LC		Z	0	0	0	2(1PaT) (1MT)	2(PaT)	0
ID_26	*Thanasimus formicarius* (Linnaeus, 1758)	LC		Z	1(BBT)	1(BBT)	0	2(PaT)	0	10(1BBT) (9MT)
				**Cryptophagidae**						
ID_27	*Pteryngium crenulatum* (Erichson, 1846)	LC		My	0	0	16(PT)	0	0	0
ID_28	*Sternodea baudii* Reitter, 1875	LC		My	0	0	1(PT)	1(PT)	0	0
				**Cucujidae**						
ID_29	*Cucujus cinaberinus* (Scopoli, 1763)	VU	NT	Z	0	0	0	7(VC)	0	0
ID_30	*Pediacus dermestoides* (Fabricius, 1792)	NT	DD	Z	0	3(PaT)	1(PT)	2(PT)	1(PaT)	1(MT)
				**Curculionidae**						
ID_31	*Hylastes ater* (Paykull, 1800)	LC		Xy	0	0	5(PaT)	4(PaT)	0	0
ID_32	*Hylastes attenuatus* Erichson, 1836	LC		Xy	0	0	12(PT)	11(10PT) (1PaT)	0	0
ID_33	*Tomicus minor* (Hartig, 1834)	LC		Xy	0	0	0	1(PT)	0	0
ID_53	*Trypodendron domesticum* (Linnaeus, 1758)	LC		My	0	0	0	0	1(PaT)	0
				**Dasytidae**						
ID_34	*Aplocnemus (Aplocnemus) nigricornis* (Fabricius, 1792)	LC		Z	0	0	5(PaT)	2(PT)	0	0
				**Elateridae**						
ID_35	*Ampedus (Ampedus) sinuatus* Germar, 1844	VU	LC	Z	0	0	4(2PT) (2MT)	2(1PaT) (1VC)	0	0
ID_36	*Melanotus (Melanotus) castanipes* (Paykull, 1800)	LC	LC	Z	0	0	4(2PaT) (2PT)	0	0	0
				**Endomychidae**						
ID_37	*Lycoperdina bovistae* (Fabricius, 1792)	LC		My	0	0	0	1(PT)	0	0
**ID**	**Species**	**IUCN**	**IUCN Eu**	**Trophic Categories**	**PF1**	**PF2**	**PF3**	**PF4**	**FB1**	**FB2**
				**Erotylidae**						
ID_38	*Dacne rufifrons* (Fabricius, 1775)	NT	DD	My	0	0	0	1(PT)	0	0
ID_39	*Triplax lacordairii* Crotch, 1870	NT	EN	My	0		2(1PaT) (1MT)	6(PT)	0	0

ID_40	*Triplax marseuli* Bedel, 1864	NT	DD	My	0	0	30(PT)	53(PT)	0	0
ID_41	*Triplax rufipes* (Fabricius, 1787)	LC	LC	My	0	0	1(MT)	1(MT)	0	0
				**Lycidae**						
ID_42	*Lygistopterus anorachilus* Ragusa, 1838	NT		My	0	0	1(PaT)	4(2MT) (2PT)	0	0
				**Melandridae**						
ID_43	*Rushia parreyssi* (Mulsant, 1856)	NT		My	0	0	0	7(PaT)	0	0
				**Melyridae**						
ID_44	*Aplocnemus* sp. Stephens, 1830			Z	0	0	0	0	1(PaT)	0
				**Monotomidae**						
ID_45	*Rhizophagus (Rhizophagus) bipustulatus* (Fabricius, 1792)	LC		My	0	0	0	0	1(MT)	0
ID_46	*Rhizophagus* sp. Herbst, 1793			My	0	0	0	0	1(MT)	0
				**Mycetophagidae**						
ID_47	*Mycetophagus quadripustulatus* (Linnaeus, 1760)	LC		My	0	1(BBT)	0	0	0	0
				**Nitidulidae**						
ID_48	*Glischrochilus quadrisignatus* (Say, 1835)	NT		My	0	0	1(PT)	1(PT)	0	0
ID_49	*Ipidia binotata* Reitter, 1875	VU		My	0	0	0	1(PaT)	0	0
ID_50	*Pityophagus ferrugineus* (Linnaeus, 1758)	LC		Z	0	0	9(PT)	11(PT)	0	0
ID_51	*Soronia grisea* (Linnaeus, 1758)	LC		SF	0	0	31(PT)	24(PT)	0	0
				**Ptinidae**						
ID_1	*Anobium punctatum* (De Geer, 1774)	LC		Xy	0	0	0	0	2(PaT)	1(PaT)
ID_2	*Anobium* sp. Fabricius, 1775			Xy	0	0	0	0	1(PaT)	1(PaT)
ID_52	*Ptinus sp.* Linnaeus, 1767			Xy	1(BBT)	1(BBT)	0	0	4(BBT)	2(BBT)
				**Staphylinidae**						
ID_54	*Anisotoma castanea* (Herbst, 1792)	LC		My	0	0	0	1(PaT)	0	0
ID_55	*Phyllodrepa salicis* (Gyllenhal, 1810)	VU		Z	0	0	1(PT)	1(PT)	0	0
**ID**	**Species**	**IUCN**	**IUCN Eu**	**Trophic Categories**	**PF1**	**PF2**	**PF3**	**PF4**	**FB1**	**FB2**
				**Tenebrionidae**						
ID_56	*Diaperis boleti* (Linnaeus, 1758)	LC		My	0	0	0	23(PT)	0	0
ID_57	*Enoplopus dentipes* (Rossi, 1790)	LC		Sx	0	0	0	13(8PT)	0	0

							(5VC)		
ID_58	*Helops caerulaeus* (Linnaeus, 1758)	LC	Sx	0	0	0	2(1PT) (1VC)	0	0
ID_59	*Nalassus dryadophilus* (Mulsant, 1854)	LC	Sx	0	0	3(PaT)	3(PaT)	0	0
				**Trogrossitidae**					
ID_60	*Grynocharis oblonga* (Linnaeus, 1757)	NT	Z	0	0	0	2(PT)	0	0
				**Zopheridae**					
ID_61	*Corticus celtis* Germar, 1824	LC	Sx	0	0	0	1(PT)	0	0
ID_62	*Endophloeus marcovichianus* (Piller&Mitterpacher, 1783)	NT	Sx	0	0	1(PaT)	0	0	0
ID_63	*Pycnomerus italicus* (Ganglbauer, 1899)	EN	Sx	0	0	0	37(27PT) (10 VC)	0	0
